# Metabolic and physical function are improved with lifelong 15% calorie restriction in aging male mice

**DOI:** 10.1007/s10522-022-09996-5

**Published:** 2022-10-31

**Authors:** Emily C. Peters, Luke Safayan, Tyler J. Marx, Emily Ngu, Anastasiia Vasileva, India Zappia, William H. Powell, Frank A. Duca, Jennifer H. Stern

**Affiliations:** 1 Department of Physiology, University of Arizona College of Medicine, Tucson, AZ 85724 USA; 2 Division of Endocrinology, University of Arizona College of Medicine, Tucson, AZ 85724 USA; 3 School of Animal and Comparative Biomedical Sciences, College of Agriculture and Life Sciences, University of Arizona, Tucson, AZ 85721 USA; 4 BIO5 Institute, University of Arizona, Tucson, AZ 85724 USA

**Keywords:** Calorie restriction, Liver fat, Glucose homeostasis, Insulin sensitivity, Healthspan

## Abstract

Chronic calorie restriction (CR) results in lengthened lifespan and reduced disease risk. Many previous studies have implemented 30–40% calorie restriction to investigate these benefits. The goal of our study was to investigate the effects of calorie restriction, beginning at 4 months of age, on metabolic and physical changes induced by aging. Male C57BL/6NCrl calorie restricted and ad libitum fed control mice were obtained from the National Institute on Aging (NIA) and studied at 10, 18, 26, and 28 months of age to better understand the metabolic changes that occur in response to CR in middle age and advanced age. Food intake was measured in ad libitum fed controls to assess the true degree of CR (15%) in these mice. We found that 15% CR decreased body mass and liver triglyceride content, improved oral glucose clearance, and increased all limb grip strength in 10- and 18-month-old mice. Glucose clearance in ad libitum fed 26- and 28-month-old mice is enhanced relative to younger mice but was not further improved by CR. CR decreased basal insulin concentrations in all age groups and improved insulin sensitivity and rotarod time to fall in 28-month-old mice. The results of our study demonstrate that even a modest reduction (15%) in caloric intake may improve metabolic and physical health. Thus, moderate calorie restriction may be a dietary intervention to promote healthy aging with improved likelihood for adherence in human populations.

## Introduction

The incidence of age-related metabolic disease, driven by fat accumulation in the liver, nearly doubles from 45 to 65 years of age (Harris et al. 1998; Cowie et al. 2009; CDC 2014, 2017). The prevalence of nonalcoholic fatty liver disease (NAFLD) rises from young adulthood to middle age, with a prevalence exceeding 40% in people over 70 years of age (Frith et al. 2009; Wang et al. 2013; Bertolotti et al. 2014). Excess hepatic lipid content is associated with impaired glucose tolerance (Borel et al. 2015) and an increased risk of developing Type II diabetes mellitus (T2DM) (Li et al. 2015; Mantovani et al. 2018) and insulin resistance (Fabbrini et al. 2009). Calorie restriction (CR), without malnutrition, is the most robust non-genetic intervention that promotes longevity and metabolic improvements to delay aging in multiple strains of laboratory rodents and non-human primates (Weindruch and Walford 1982; Weindruch et al. 1986; Anderson et al. 2009). Ten years of 30% CR in rhesus monkeys decreases body fat, improves insulin sensitivity, and decreases circulating insulin (Gresl et al. 2003). Lifelong 40% CR decreases liver fat and the expression of lipogenic genes at the liver in 18-month-old mice (Kuhla et al. 2014). Lifelong 30% CR decreases liver fat, improves glucose clearance, and lowers circulating insulin levels in 12-month-old mice (Rusli et al. 2015).

The majority of CR studies in rodents impose a degree of caloric restriction (~ 30–40%) (Kuhla et al. 2014; Karunadharma et al. 2015; Gutierrez-Casado et al. 2019; Mezhnina et al. 2020) that would be difficult to maintain. A major challenge in translating CR studies in rodents to humans is the severity of CR. In fact, although human participants in the 2-years CALERIE™ (Comprehensive Assessment of Long-Term Effects of Reducing Intake of Energy) phase 1 trial were asked to restrict caloric intake by 25% for the 2-years duration of the study, in practice the participants actually achieved less than half that level of restriction (11.9%) in daily caloric intake (Kraus et al. 2019). For the aging field to make strides in translating studies of CR in model organisms to that of humans, it is critical to evaluate the effect of a more modest calorie restriction regimen in promoting healthy aging. As a species that shares the majority of its protein-coding genes with humans, the mouse serves as an excellent model to study interventions that may affect human aging and disease (Yue et al. 2014). We evaluated the metabolic effects of lifelong moderate (15%) calorie restriction, initiated at 4 months of age, in 10-, 18-, 26-, and 28-month-old mice. Studying mice at these ages allows us to examine the effects of moderate CR at ages that are equivalent to middle age (10 months), the lower (18 months) and upper limits (26- and 28-months) of old age in humans (Flurkey et al. 2007).

## Materials and methods

### Animals

**Fig. 1:**
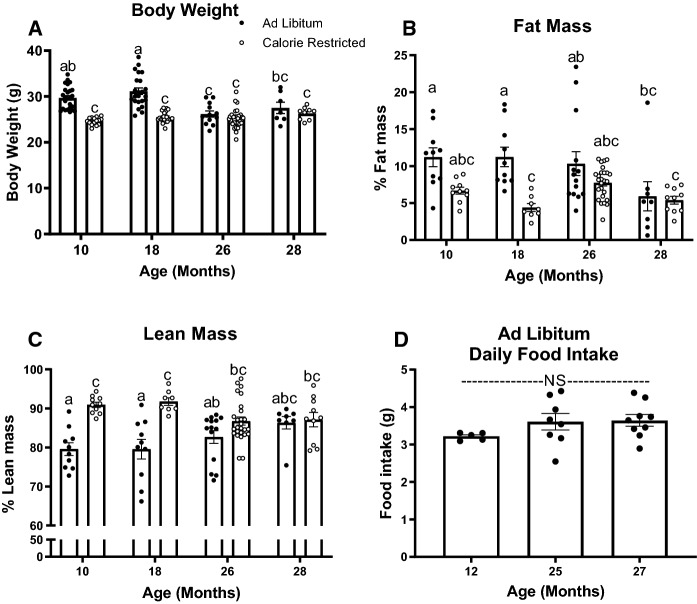
Body weight, body composition, and ad libitum food intake. Body weight (**A**) of ad libitum and calorie-restricted mice at 10 (*n* = 15–25), 18 (*n* = 23–25), 26 (*n* = 12–34), and 28 months of age (*n* = 7–9). Fat mass percentage (**B**) and lean mass percentage (**C**) of 10- (*n* = 10), 18- (*n* = 8–10), 26- (*n* = 14–26), and 28-month-old AL and CR mice (*n* = 8–10). Ad libitum daily food intake (**D**) was measured for 7 consecutive days in 12- (*n* = 5), 25- (*n* = 8), and 27-month-old mice (*n* = 9). Each data point represents the average daily food intake of one mouse. Data presented as Mean ± SEM; ^a,b,c^Superscript letters that differ indicate differences, *P* < 0.05; Figures **A**–**C**: two-way ANOVA with Tukey’s adjustment for multiple comparisons. Figure **D**: One-way ANOVA with Tukey’s adjustment for multiple comparisons, NS (not significant)

All animal procedures in this study were approved by the Institutional Animal Care and Use Committee of the University of Arizona College of Medicine (IACUC protocol 18-478). All experimental procedures were performed according to NIH guidelines. Male C57BL/6NCrl mice were obtained from the National Institute on Aging caloric restriction colony (Charles River, Wilmington, MA). Mice were singly housed and maintained on a 14-h light/10-h dark cycle. Ad libitum-fed mice had unlimited access to food (NIH-31), while calorie-restricted mice received 85% (3 g of NIH-31) of their ad libitum food intake in tablet form (LabDiet Cat# 1819591-300) once daily at 6 p.m., the onset of the dark cycle. The level of CR was calculated as a function of daily food intake assessed in ad libitum mice (Fig. 1D).

### Oral glucose tolerance test

10-, 18-, 26-, and 28-month-old mice were fasted for 4 h prior to oral gavage of D-glucose (2.5 g/kg; Fisher). Blood was collected by tail nick, and blood glucose was assessed at baseline and 15, 30, 60, 90, and 120 min after oral glucose was administered with a glucometer (9556c, Bayer, Leverkusen, Germany). Blood was collected at baseline and 15 min after gavage to assess basal and oral glucose-stimulated serum insulin.

### Insulin tolerance test

To assess differences in insulin sensitivity in advanced age, 28-months ad libitum and calorie restricted mice were injected intraperitoneally with insulin (0.25 IU/kg body weight) after a 4-h fast. Blood glucose concentration was assessed using glucometer at baseline and 15, 30, 60, 90, and 120 min after insulin injection. Tail blood was collected at baseline and 15 min after insulin injection to assess hypoglycemia-stimulated glucagon secretion.

### In vivo assessment of physical function

To assess the impact of aging and caloric restriction on forelimb and all limb grip strength (Justice et al. 2014), we allowed each mouse to grip a metal wired pad attached to a force transducer (San Diego Instruments, San Diego, CA) with forepaws only or forepaws and hind paws. We then pulled the mouse horizontally and measured force until its grip was released. Forelimb and all limb grip strength was tested in 5 trials/day. The minimum and maximum force values were omitted, then the remaining three measures were used to calculate average grip strength. Average grip strength was normalized to body weight to account for differences in body weight with calorie restriction.

To assess balance and coordination in 26- and 28-month-old mice, we performed a rotarod task using a continuous-acceleration apparatus (Columbus Instruments, Columbus, OH) Mice were placed on a stationary rod and were given the chance to stabilize their posture before each trial, which consisted of incremental rod acceleration starting at 4 rotations per minute (rpm) and increasing by 0.5 rpm every 5 s. The main outcome measured was time to fall (seconds). Each mouse was tested in 3 trials/day and each data point represents the average time to fall per mouse (Justice et al. 2014).

### Body weight and body composition

Body weights were measured after a 4 h fast. Fat mass and lean mass was assessed via NMR (EchoMRI™, Houston, TX). Percent of fat mass or lean mass was based off total body weight.

### Tissue collection

Mice were sacrificed at 10, 18, 26, and 28-months of age. Mice were fasted for four hours, (9 a.m.–1 p.m.) then anesthetized by bell jar isoflurane exposure and immediately decapitated. We collected trunk blood and allowed the blood to clot on ice for 30 min. We collected serum after centrifugation at 3000×*g* for 20 min at 4 °C. Aliquots were stored at − 80 °C until analysis. Tissues were snap frozen on dry ice and stored at − 80 °C until analysis.

### Hepatic lipid content

We powdered the livers using a liquid nitrogen cooled mortar and pestle. 10–15 mg of frozen, powdered liver was weighed and sonicated in 100 µL PBS, then 1 mL of 100% ethanol was added to each sample. Samples were vortexed for 20 min then centrifuged at 16,000×*g* at 4 °C (Geisler et al. 2019; Vasileva et al. 2022). Supernatant was transferred to a fresh tube for analysis of liver triglycerides (TAG) (Cat. # T7531, Pointe Scientific Inc., Canton, MI) and non-esterified fatty acids (NEFA) (FUJIFILM Wako Pure Chemical Corporation). Total hepatic triglyceride content was calculated as mg/g tissue and total hepatic NEFA content was calculated as µmol/g tissue.

### RNA isolation and RT-qPCR

RNA was extracted from powdered livers using TRIzol™ Reagent. (Thermo Fisher Scientific, Waltham, MA). Extracted RNA was washed with water-saturated butanol and ether to eliminate phenol (Krebs et al. 2009). Reverse transcription was performed using Verso cDNA synthesis kits (Thermo Fisher Scientific, Waltham, MA), and RT-qPCR was performed using SsoAdvanced Universal SYBR^®^ Green Supermix (Bio-Rad Laboratories, Hercules, CA) on the Applied Biosystems QuantStudio 6 Flex Real-Time PCR System (Applied Biosystems™, Foster City, CA). Raw Ct values were analyzed using LinReg PCR analysis software to determine amplification efficiency (Ramakers et al. 2003). Genes of interest were normalized to *β-actin* expression and the fold change in gene expression was calculated using the efficiency^∆∆Ct^ method (Livak and Schmittgen 2001). Fold change for all age and diet groups was calculated against the ad libitum-fed 10-month-old group. Mouse primer sequences for all genes analyzed with real-time PCR are presented in Table 1.

**Table 1:** List of primer sequences for RT-PCR

Gene	Forward primer (5′–3′)	Reverse primer (5′–3′)	Gene ID
Mouse *Actb*	TCGGTGACATCAAAGAGAAG	GATGCCACAGGATTCCATA	11461
Mouse *Acaca*	ATGGGCGGAATGGTCTCTTTC	TGGGGACCTTGTCTTCATCAT	107476
Mouse *Acly*	CAGCCAAGGCAATTTCAGAGC	CTCGACGTTTGATTAACTGGTCT	104112

### Serum analyses

We measured serum insulin and glucagon concentrations using commercially available enzyme-linked immunosorbent assays **(**Insulin: Cat. # 80-INSMSU-E10, Alpco, Salem, NH; Glucagon: Cat. # 10-1281-01, Mercodia, Uppsala, Sweden). HOMA-IR index was calculated according to the formula: HOMA-IR = fasting glucose in mmol/l*fasting insulin in μU/mL/22.5 (Sarafidis et al. 2007).

We assessed serum triglyceride (TAG), non-esterified fatty acids (NEFA), and glucose concentrations using colorimetric assays (Glucose: Cat. # G7521, Pointe Scientific Inc., Canton MI; NEFA: FUJIFILM Wako Pure Chemical Corporation; TAG: Cat. # T7532, Pointe Scientific Inc., Canton, MI).

### Statistical analyses

We performed statistical analyses in SAS Enterprise Guide 7.1 (SAS Institute Inc., Cary, NC) and GraphPad Prism Version 9.4.0 (GraphPad Software, San Diego, California, USA). We used two-way ANOVA to assess the effect of aging and 15% caloric restriction on all dependent variables. The probability of difference between means was assessed after a Tukey’s adjustment for multiple comparisons. Glucose-stimulated serum insulin and hypoglycemia stimulated serum glucagon were analyzed using paired *t*-tests to assess the change in serum hormone concentration between two timepoints within each mouse. Raw data were plotted in GraphPad Prism Version 9.4.0 for Windows (GraphPad software). All data are presented as mean ± SEM.

## Results

### Lifelong moderate CR decreases body weight and fat mass in 10- and 18-month-old mice

We first sought to assess how a moderate calorie restriction affects body weight and body composition in middle-aged and old-aged mice. We found that in 10- and 18-month-old mice, 15% calorie restriction resulted in significantly lower body weight compared to ad libitum controls (Fig. 1A , *P* < 0.0001). At 26 and 28 months of age, there was no significant difference in body weight between ad libitum and calorie restricted mice. In ad libitum fed mice, body weight did not differ from 10 and 18 months of age but was decreased in 26- and 28-month-old mice.

We assessed body composition using EchoMRI and found that CR decreased percent fat mass in 18-month-old mice (Fig. 1B, *P* < 0.01). CR increased percent lean mass at 10- and 18-months of age (Fig. 1C, *P* < 0.001).

### Lifelong moderate CR decreases serum insulin without affecting serum TAG or NEFA

**Fig. 2:**
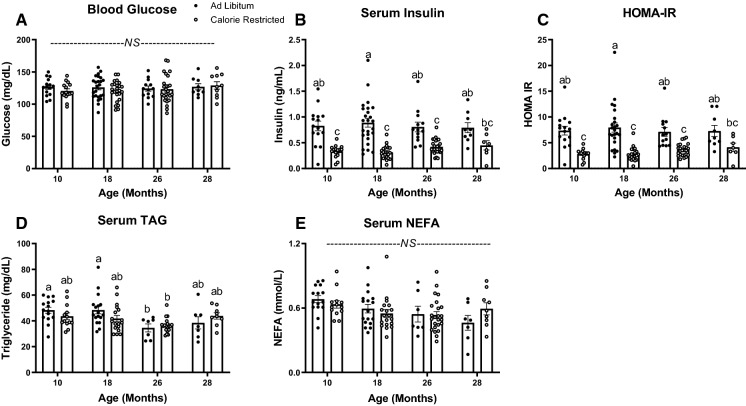
4-h fasted blood glucose, serum insulin, Homeostatic Model Assessment of Insulin Resistance (HOMA-IR), serum triglyceride (TAG), and non-esterified fatty acids (NEFA) in ad libitum-fed compared to 15% Calorie Restricted (CR) mice. Blood glucose (**A**) at 10 (*n* = 15), 18 (*n* = 24–25), 26 (*n* = 13–26), and 28 months of age (*n* = 9–10). Serum insulin (**B**) and HOMA-IR (**C**) at 10 (*n* = 15), 18 (*n* = 21–25), 26 (*n* = 13–25), and 28 months of age (*n* = 7–9). **C** Serum TAG at 10 (*n* = 13–15), 18 (*n* = 17–18), 26 (*n* = 7–19), and 28 months of age (*n* = 7–9) and **D** serum NEFA at 10 (*n* = 15–13), 18 (*n* = 17–19), 26 (*n* = 7–24), and 28 months of age (*n* = 7–9). Data presented as Mean ± SEM; ^a,b,c^Superscript letters that differ indicate differences, *P* < 0.05; two-way ANOVA with Tukey’s adjustment for multiple comparisons; NS (not significant)

We next assessed circulating glucose, insulin, TAG, and NEFA concentrations after a 4 h fast. Neither age, nor CR had an effect on blood glucose concentration (Fig. 2A). Despite similar glucose concentrations, CR decreased serum insulin concentration in 10-, 18-, and 26-month-old mice (*P* < 0.001, Fig. 2B), suggestive of improved insulin sensitivity. In line with this finding, HOMA-IR, an indicator of insulin resistance (Sarafidis et al. 2007), decreased in response to CR across mice of all ages (*P* < 0.001, Fig. 2C). Because serum insulin and insulin resistance are often correlated with elevated circulating NEFA and TAG in adults (Frohnert et al. 2013), we assessed the effects of both age and CR on serum NEFA and TAG. While neither age, nor CR affected serum NEFA, we found that serum TAG concentration was lower only in ad libitum fed 26-month-old mice compared to 10- or 18-month-old ad libitum fed mice. CR did not affect serum TAG, regardless of age (Fig. 2D, E, *P*  < 0.05).

### Lifelong moderate CR decreases hepatic triglyceride at 10 and 18 months of age

**Fig. 3:**
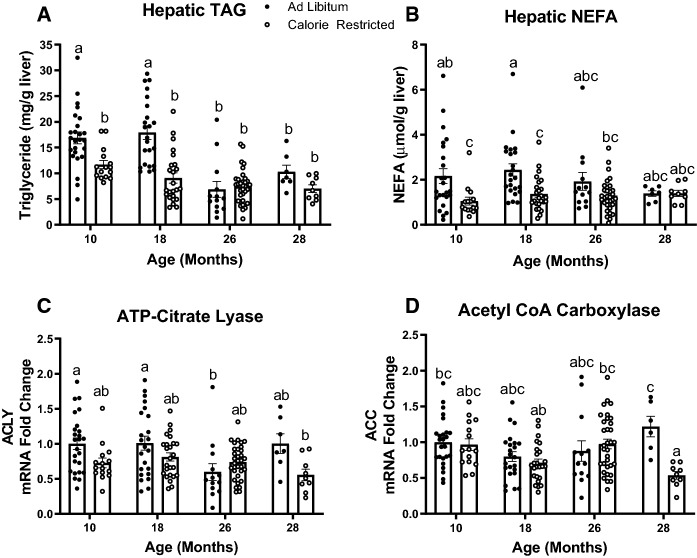
Hepatic lipid accumulation and lipogenic gene expression ad libitum-fed compared to 15% Calorie Restricted (CR) mice. **A** Liver triglyceride content in 10- (*n* = 15–25), 18- (*n* = 23–24), 26- (*n* = 13–33), and 28-month-old AL and CR mice (*n* = 7–9). **B** Liver non-esterified fatty acids (NEFA) in 10- (*n* = 15–24), 18- (*n* = 23–25), 26- (*n* = 13–33), and 28-month-old mice (*n* = 7–9). Liver mRNA fold change of (**C**) ATP-citrate lyase and **D** acetyl CoA carboxylase in 10- (*n* = 15–25), 18- (*n* = 23–25), 26- (*n* = 13–33), and 28-month-old mice (*n* = 7–9). Gene expression was normalized to the housekeeping gene ACTβ and presented as relative expression compared to the 10-month-old ad libitum fed group. ^a,b,c^Letters that differ indicate differences within group, *P* < 0.05; two-way ANOVA with Tukey’s adjustment for multiple comparisons. Data presented as Mean ± SEM

We next assessed the impact of 15% CR on hepatic lipid content in 10-, 18-, 26-, and 28-month-old mice. We found that this moderate level of CR decreased liver TAG and NEFA content in 10- and 18-month-old mice (*P* < 0.01), but not at 26 or 28 months of age. Hepatic TAG concentrations decreased with advanced age (26- and 28-months of age, *P* < 0.05) in ad libitum fed mice. We observed no age effect on liver NEFA (Fig. 3A, B).

As a first step to better understand the CR and age effects on hepatic lipid content, we next assessed liver ATP citrate lyase (ACLY) and acetyl CoA carboxylase (ACC) mRNA expression. These are two key enzymes involved in hepatic de novo lipogenesis (Thampy and Wakil 1988; Kim et al. 2017). CR did not affect mRNA expression of ACC or ACLY at 10-, 18-, or 26- months of age (Fig. 3C, D). However, CR decreased ACC mRNA expression at 28 months of age (Fig. 3D, *P*  < 0.01).

### Lifelong moderate CR improves glucose clearance at 10 and 18 months of age

**Fig. 4:**
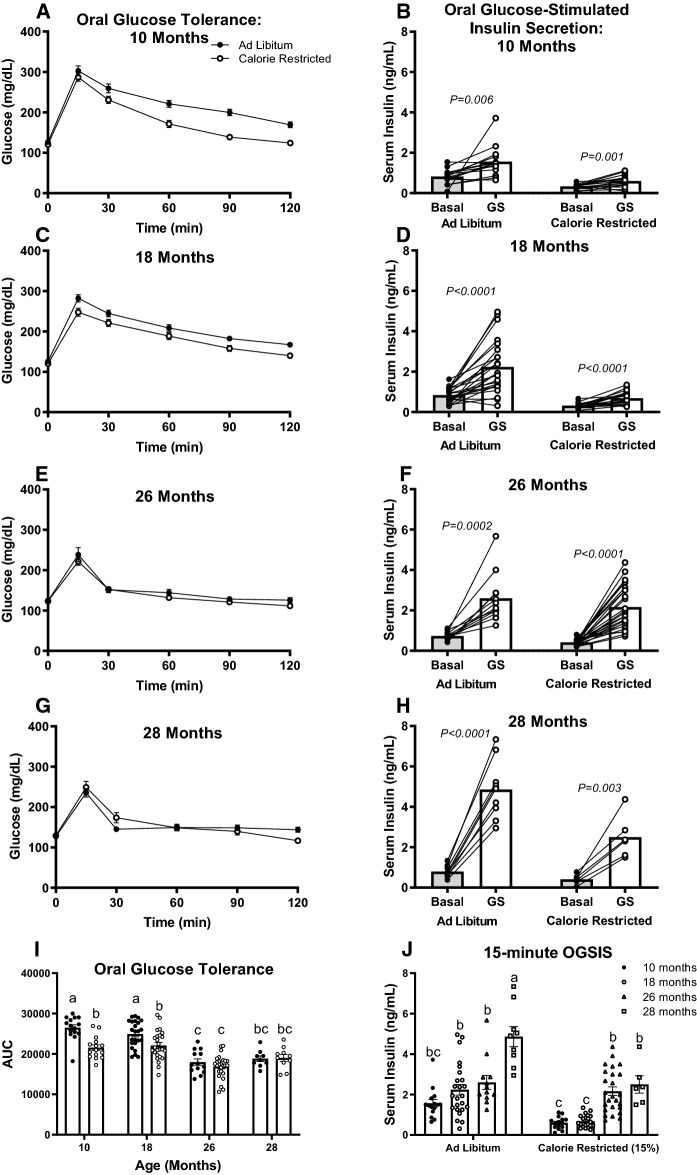
Oral glucose clearance and oral glucose-stimulated insulin secretion (OGSIS). Blood glucose curves (**A**, **C**, **E**, and **G**) and the area-under-the-curve (**I**) from oral glucose tolerance tests in ad libitum fed and 15% Calorie Restricted (CR) mice at 10 (*n* = 15), 18 (*n* = 24–25), 26 (*n* = 13–26), and 28 months of age (*n* = 9–10). ^a,b,c^Letters that differ indicate differences, *P* < 0.05; two-way ANOVA with Tukey’s adjustment for multiple comparisons. Oral glucose-stimulated insulin secretion (OGSIS) (**B**, **D**, **F**, **H**) from baseline to 15 min after oral glucose (2.5 g/kg BW) gavage (10 months: *n* = 15; 18 months: *n* = 21–24; 26 months: *n* = 12–25; 28 months: *n* = 6–9). *P* values reflect results of paired samples *t* tests to assess the change in serum insulin concentration between timepoints within each mouse (**B**, **D**, **F**, **H**). Effect of aging on OGSIS 15-min-post oral glucose gavage (**J**); ^a,b,c^Letters that differ indicate differences, *P* < 0.05; two-way ANOVA with Tukey’s adjustment for multiple comparisons. Data presented as Mean ± SEM

Because hepatic lipid accumulation is tightly coupled to glucose homeostasis (Borel et al. 2015; Lomonaco et al. 2016), we next performed oral glucose tolerance tests (OGTT) to assess the effects of calorie restriction on glucose clearance and oral glucose-stimulated insulin. Moderate calorie restriction improved oral glucose clearance in 10- and 18-month-old mice, the same mice in whom calorie restriction had lowered liver lipid concentration (Fig. 4A, C and I, *P* < 0.0001). Interestingly, coinciding with decreased liver triglycerides, glucose clearance was improved in ad libitum fed 26- and 28-month-old mice relative to 10- and 18-month-old mice. CR did not further improve glucose clearance in 26- and 28-month-old mice compared to ad libitum fed age-matched controls (Fig. 4E, G and I).

Having demonstrated that CR improves glucose clearance in 10- and 18-month-old mice and that glucose clearance improves in advanced age, we sought out to determine if these improvements were a result of changes in oral glucose stimulated insulin secretion (OGSIS). We assessed serum insulin concentrations at baseline and 15 min after oral glucose was administered. In each age and diet group, oral glucose gavage increased serum insulin concentrations 15 min after administration (Fig. 4B, D, F, H). CR decreased OGSIS only at 18 and 28 months of age. Of note, aging increases oral glucose stimulated serum insulin in both ad libitum and CR mice (*P* < 0.001, Fig. 4J).

### Lifelong moderate CR increases insulin sensitivity in 28-month-old mice

**Fig. 5:**
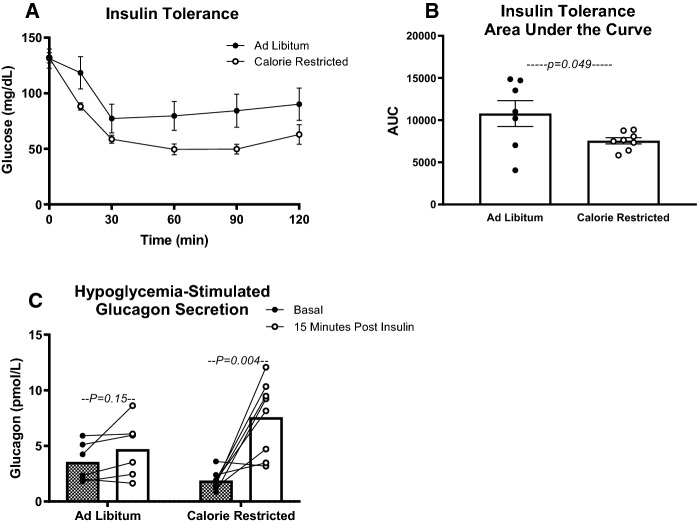
Lifelong 15% calorie restriction improves insulin sensitivity in advanced age. Blood glucose curves (**A**) and area-under-the-curve (**B**) from insulin tolerance tests in 28-month-old ad libitum fed and 15% calorie restricted (CR) mice (*n* = 7–8); *P* values reflect results of unpaired t-tests to assess difference between diet groups. **C** Serum glucagon concentrations at baseline and 15 min after intraperitoneal insulin injection (0.25 IU/kg BW) (*n* = 6–8); *P* values reflect results of paired samples *t* tests to assess the change in serum insulin concentration between timepoints within each mouse. Data presented as Mean ± SEM

Having established the effect of aging and CR on glucose clearance and OGSIS, we sought out to assess the effect of CR on insulin sensitivity in mice of advance age (28 months of age). Although basal glucose was nearly identical in both groups of mice, insulin more severely decreased blood glucose concentration in calorie restricted mice (Fig. 5A and B). We next collected tail blood at 0 and 15 min during the ITT to assess the effect of calorie restriction on hypoglycemia-stimulated glucagon secretion. Glucagon, the counterregulatory hormone to insulin, increases in response to hypoglycemia and extended fasting to promote hepatic glycogenolysis and increase blood glucose (Stern et al. 2019; Vasileva et al. 2022). While serum glucagon did not change from 0 to 15 min after insulin injection in ad libitum* mice*, we observed a robust rise in serum glucagon in the calorie restricted group (*P* = 0.004, Fig. 5C). This more robust rise in serum glucagon would be expected to limit the apparent insulin sensitivity measured by ITT. In turn, the CR induced improvement in insulin sensitivity is likely more robust than suggested by the ITT.

### Lifelong moderate CR improves physical function

**Fig. 6:**
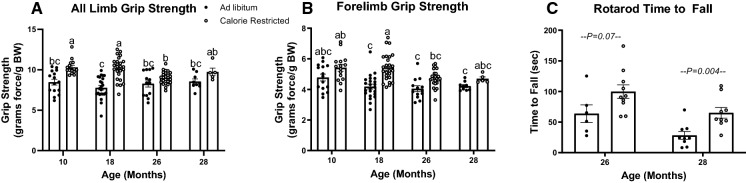
In vivo measures of physical function. All limb (**A**) and forelimb (**B**) grip strength in ad libitum fed and 15% calorie restricted mice at 10 (*n* = 15), 18 (*n* = 23–25), 26 (*n* = 13–24), and 28 (*n* = 5–9) months of age; ^a,b,c^Letters that differ indicate differences, *P* < 0.05; two-way ANOVA with Tukey’s adjustment for multiple comparisons (**A**, **B**). Rotarod task was performed in 26- (*n* = 6–10) and 28-month-old mice (*n* = 9); *P* values reflect results of unpaired t-tests to assess difference between diet groups. Data presented as Mean ± SEM

Because metabolic health correlates with physical function (Fritschi et al. 2017), we next assessed the effects of caloric restriction and aging on measures of physical function. We performed grip strength tests and found that calorie restriction improved all limb grip strength at 10- and 18-months of age and forelimb grip strength at 18-months of age (Fig. 6A  and B, *P* < 0.001 and *P* < 0.0001, respectively), but we saw no effect of CR on grip strength at 26 or 28 months of age. To assess balance and coordination, we performed the rotarod test in 26- and 28-month-old mice. Calorie restriction tended to increase the time to fall in 26-month-old mice (*P* = 0.07, Fig. 6C) and significantly increased time to fall in 28-month-old mice (Fig. 6C, *P* = 0.004).

## Discussion

In the present study, we sought to investigate the effects of lifelong moderate (15% initiated at 4 months of age) CR on hepatic lipid accumulation, glucose homeostasis, and physical function at timepoints that include stages representative of middle to advanced age (10-, 18-, 26, and 28-months) in mice. A more severe (30–40%) level of restriction has been the focus of most studies that aim to assess CR’s effect on metabolic function in animal models of aging. Still, few studies have investigated the metabolic impact of this restriction in advanced age. Lifelong 30% CR decreases liver fat, improves glucose clearance, and lowers circulating insulin levels in 12-month-old mice (Rusli et al. 2015). 40% CR initiated at 3 months decreases liver fat at 12- and 15-months of age (Ogrodnik et al. 2017). Similarly, 74 weeks of 40% CR (fed at 8 A.M.) decreases liver fat at 19 months of age (Kuhla et al. 2014).

We found that a modest lifelong 15% CR decreased body mass and fat mass in 10- and 18-month-old mice, but not at 26 and 28 months of age (advanced age). Our observation that CR did not further decrease body weight or fat mass compared to age-matched ad libitum fed mice is likely due to the decrease in body weight commonly seen during advanced age in ad libitum fed C57Bl/6 mice, preventing us from observing a further decrease in body weight or fat mass. Supporting these findings, Turturro and colleagues also observed an age-related decrease in body mass beginning at approximately 25 months of age, which continued to decline throughout advanced age (Turturro et al. 2002).

CR decreased liver triglyceride content in 10- and 18-month-old mice and, accordingly, improved glucose tolerance at these ages. In advanced aged (26 and 28 months) mice, liver fat was decreased compared to 10- and 18-month-old mice, and CR did not further decrease liver triglyceride, liver NEFA, or affect glucose clearance at these ages (Figs. 3A, B and 4I). Despite the key role of hepatic lipid content on metabolic flux, few studies have extended beyond 22 months of age to investigate the effect of aging on hepatic lipid concentration. Fontana and colleagues showed that liver triglyceride content was similar in 6-, 12-, and 22-month-old C57BL/6 mice fed either a low-fat chow or high fat diet (Fontana et al. 2013). Our observation that hepatic lipid accumulation decreases in advanced age is likely secondary to the age-related decrease in body weight and fat mass. The age dependent decrease in hepatic lipid accumulation in 26- and 28-month-old mice likely prevents us from observing any benefits from CR, a treatment aiming to decrease liver lipid and improve metabolic health.

Given the decrease in liver triglyceride content that we observed with both calorie restriction and aging, we performed qPCR to evaluate the mRNA expression of two crucial enzymes in the de novo lipogenesis pathway, ATP-citrate lyase (*Acly* or ACLY) and acetyl CoA carboxylase (*Acaca* or ACC). ACLY generates acetyl CoA, which is the substrate for ACC, the rate limiting and first committed step in de novo lipogenesis (Thampy and Wakil 1988). Pharmacologic and genetic inhibition of both ACLY (Wang et al. 2009) and ACC (Kim et al. 2017) decreases hepatic lipid accumulation. We did not observe robust changes in liver ACC or ACLY gene expression in response to either aging or calorie restriction, suggesting that transcriptional regulation of these genes is not robustly affecting hepatic lipid changes that result from calorie restriction or aging (Fig. 3C, D). Still, post-translational modification does robustly affect activity of acetyl CoA carboxylase. The activity of acetyl CoA carboxylase is inhibited by phosphorylation via AMP-activated protein kinase (AMPK) (Garcia et al. 2019; Lally et al. 2019) in response to a rise in the AMP:ATP ratio when cellular energy levels are low. Acetyl CoA carboxylase is regulated by the glucoregulatory hormones insulin and glucagon, encouraging lipid production when food is available and inhibiting lipid production when food is scarce. Hepatic insulin resistance in response to aging may decrease liver lipid content (Brown and Goldstein 2008).

Interestingly, we found that glucose tolerance improved with advanced age (26 and 28 months old; Fig. 4I). Yet, there was no further improvement in glucose clearance with CR in mice of advanced age. This observation is in stark contrast with what has been observed in aging humans (Shimokata et al. 1991; Ehrhardt et al. 2019). Typically, as humans age from middle to advanced age, glucose tolerance decreases. In fact, analyses from over 700 participants in the Baltimore Longitudinal Study of Aging, show that glucose tolerance declines from 60 to 92 years of age, independent of changes in body composition and activity levels (Shimokata et al. 1991). We found that OGSIS increases in advanced age in mice (Fig. 4J), possibly explaining the improved glucose clearance. Thus, aged mice secreted higher levels of insulin in order to clear blood glucose. In line with our findings, Oh and colleagues (2016), studying mice from 4 to 20 months of age, found that aging did not affect blood glucose concentrations, but improved glucose tolerance while decreasing insulin sensitivity (Oh et al. 2016). They similarly showed that aging (20 months) increased glucose-stimulated serum insulin (Oh et al. 2016). Using HOMA-IR to assess insulin resistance, we found that insulin sensitivity was similar at all ages 10–28 months of age (Fig. 2C). Importantly, 15% calorie restriction decreased HOMA-IR across mice of all ages (Fig. 2C).

Dysregulated glucose and lipid homeostasis increases the risk of developing limitations in physical function in older persons (Penninx et al. 2009). Aging causes a decline in physical function that can be delayed by caloric restriction. Similar to studies that implement a more severe level of restriction (Orenduff et al. 2022), the 15% calorie restriction we implemented improved forelimb and all limb grip strength in most age groups and improved balance and coordination in 26- and 28-month-old mice, as measured by time to fall during the Rotarod task. Grip strength measurements were normalized to body weight, and calorie restricted mice had a higher percentage of lean mass per gram body weight, thus it is reasonable that calorie restricted mice had a greater normalized grip strength than ad libitum mice. However, the increased time to fall during the Rotarod task is not corrected by body mass.

We must be judicious in raising the limitations of translating data from rodent models of caloric restriction to human aging and metabolic health. Observational studies of humans that involuntarily restrict caloric intake propose that there may be maintainable beneficial effects of modest calorie restriction. Based on six decades of archived dietary intake data, Willcox and colleagues (2007) estimated that residents of Okinawa self-impose approximately an 11% caloric restriction. This correlated with a life-long low BMI, decreased mortality from age-associated diseases, and extended mean and maximum lifespan (Willcox et al. 2007). Although promising, these correlative findings do not demonstrate direct causation between modest caloric restriction and increased lifespan in humans. Long term clinical trials are first required to assess the efficacy of moderate CR in preventing age-related disease and improving healthspan. While few controlled human trials have examined the physiological effects of long-term CR, data generated from the CALERIE trial (Comprehensive Assessment of Long-Term Effects of Reducing Intake of Energy) supports the hypothesis that there are substantial beneficial effects of modest CR. A 12% calorie restriction decreased body weight, fat mass (Das et al. 2017), and reduced multiple cardiometabolic risk factors, including LDL cholesterol, total: HDL cholesterol, and insulin sensitivity, independent of weight loss (Kraus et al. 2019). These encouraging findings from the CALERIE™ trial and our data from similarly calorie restricted (15%) mice support the need for future research aimed at understanding the metabolic impact of moderate caloric restriction in both human populations and animal models of aging.

There are some limitations of our study that must be considered when interpreting results. One potential limitation of our study is the variable fasting durations between our ad libitum and calorie restricted mice for in vivo metabolic studies and tissue collections. Ad libitum fed mice would have been imposed with a 4 h fast (likely ate very little for 8 h, since lights on), while calorie restricted mice likely fasted for 14–16 h. This limitation is hard to overcome, as feeding the daily food allotment hours prior to sacrifice would potentially have a greater impact on our measures of metabolic health. Ideally, we would have performed insulin tolerance tests on all ages of mice. However, HOMA-IR does provide a measure of insulin resistance, establishing that calorie restriction improves insulin sensitivity across all ages of mice. We recognize that the number of mice in the 28-month-old age group is low, relative to younger age groups in this study. The median lifespan of ad libitum fed C57Bl/6 mice is approximately 28 months (Turturro et al. 2002). Hence this discrepancy in the number of mice per age group was unavoidable due to mortality at this advanced age. Another limitation is that our studies are limited to male mice. Hormonal changes that occur in midlife in women are associated with dysregulation of lipid (Fan and Dwyer 2007; Derby et al. 2009; Woodard et al. 2011) and glucose (Lindheim et al. 1994; Ryan et al. 2002; Rossi et al. 2004) homeostasis. Similarly, mouse models of menopause exhibit weight gain, elevated fasting insulin, and insulin resistance (Romero-Aleshire et al. 2009). Given these metabolic consequences that occur during midlife that are unique to women, studies examining the effects of moderate caloric restriction in female mice across the lifespan is essential to understand potential sex differences in the metabolic response to caloric restriction.

Our findings indicate that a moderate, maintainable level of calorie restriction beginning at early adulthood can limit the decline in metabolic and physical (strength, balance, and coordination) function with aging in mice. In conclusion, 15% calorie restriction may cause comparable metabolic and physical benefits to the typical higher percentage CR, with the added benefit of increased likelihood of compliance in human populations. These findings support the need for future research aimed at understanding the physiological impact of modest caloric restriction in animal models of aging.

## Data Availability

The datasets generated during and/or analyzed during the current study are available from the corresponding author on reasonable request.
